# Report of the Committee Appointed under the Fourth Resolution of the National Medical Convention, Which Assembled in New York, in May, 1846

**Published:** 1847-08

**Authors:** Ro. W. Haxall

**Affiliations:** Chairman


*Report of the Committee appointed under the fourth resolution of the National Medical Convention, which assembled in New York, in May*, 1846.

*Resolved*, —That it is desirable that a uniform and elevated standard of requirements for the degree of M. D. should be adopted by all the Medical Schools in the United States; and that a committee of seven be appointed to report on this subject, at a meeting to be held in Philadelphia on the first Wednesday in May, 1847.

*Committee*—Drs. Haxall and Cullen, of Richmond; S. A. Patteson, Manchester, Va,; A. Flint, Buffalo, N. Y.; J. Perkins, Castleton, Vt.; J. A. Wing, Albany, N. Y.; Geo. W. Norris, Philadelphia.

The committee under the fourth resolution of the convention which assembled in New York in May, 1846, and which has for its object the adoption of a uniform and elevated standard of requirements for the degree of M. D., have had the same under consideration, and beg leave to offer the following

REPORT.

The excitement which appears to have pervaded the medical mind for some considerable time in relation to the subject of reform, not only in this but in foreign countries, would of itself present an argument sufficiently demonstrative of its importance.

The ceaseless and unwearied exertions of the master-minds of the profession, constantly urging on improvement in the various departments of medicine, have elaborated a work which it will require years of toilsome study justly to appreciate; and while we are far from indulging the Utopian idea that the newly graduated student is to be immediately transformed into the accomplished physician, yet do we believe from the complaints which spring from almost every quarter, that lamentable deficiencies do exist, and that these deficiencies should be corrected.

Nor do we stand alone in this opinion. The same spirit of reform which has assembled this convention, produced in Great Britain amendments to the charters of her Colleges, and convoked the Medical Congress which sat in Paris, than which a more renowned body of men never met to deliberate upon the interests of the profession, Not a great deal, it is true, has yet been accomplished in Great Britain, owing to the influence still exercised by medical corporations, and the indifference with which Parliament has regarded the efforts of the reformers. But in enlightened France, protected and cherished as are all her literary institutions by government, the most propitious results have been obtained. The term of study has been prolonged; other branches of medical education have been added to the already extensive curriculum, and more frequent and rigid examinations have been adopted by which to test the qualifications of the candidate.

And when we consider the existing systems of other countries, how vast a difference is manifested upon the most cursory examination; truly must it be acknowledged that the mass of our medical graduates is inferior to those of other climes, or that the talent of America, like its broad and impetuous rivers, or its majestic forests, can recognize no equality in others.

We are free to confess that within the last twenty or five-and-twenty years, medicine has been better taught among us than at any former period. It is difficult to imagine, indeed, that the progressive efforts of foreign lands should pass wholly unnoticed in this; and, while their example has been productive of some improvement, much yet remains to be accomplished. It is unquestioned that the facilities for education have been augmented not only in the public schools of the country, but private enterprise has exerted no slight influence in the attainment of this end. But let us not remain stationary while the interests of society and the profession impel us to further advancement.

If we discard the preliminary education demanded of students in Europe, and confine our attention to the branches actually taught in the medical schools, it will be perceived that the deficiency on our part is sufficiently apparent. All the branches which we profess to teach are taught by them; in addition to which (and now we refer more particularly to continental institutions) we may enumerate the several subjects of Zoology, Botany, Comparative Anatomy, the History of Medicine, Hygiene, Medical Physics, and Clinical Medicine and Surgery. All these branches, it is true, are not crowded upon the mind during a single session, but are distributed throughout successive years; while clinical instruction, in both medicine and surgery, is reserved for the last year or two, after it is presumed the student has made such advances in his primary studies as will enable him perfectly to understand the lessons which are taught him at the bedside of the patient.

Instruction so full and so complete must, of necessity, require a lengthened period for its accomplishment; hence we find that, in France and Austria, the term of lectures is extended to a fraction more than four years; in Germany to three years and four months; in Great Britain to two years, while in the schools of our own country, two sessions of four months each are alone sufficient to entitle the candidate to an examination. And if we extend our inquiries a step further, we shall find as great a contrast in regard to the examinations by which the qualifications of candidates are tested. Nowhere, we believe, except in England and America, is the qualification made to rest upon a single viva-voce examination; but throughout the period of the student's probation, repeated examinations are held at stated intervals. By proceeding in this manner, not only is the qualification more accurately ascertained, but certain branches are completely mastered before others have been commenced. Thus we learn from Mr. Surgeon Wilde's work on Austria and her institutions, that, at Vienna, the student is compelled to undergo an examination at the end of every six months by each professor whose lectures he may have attended. During the fifth year, which completes his term of study, he is subjected to two other examinations, besides giving a detailed history of two or more clinical cases, and defending in public a thesis written in the Latin language. In Prussia, four examinations are required. When the pupil has satisfactorily completed the third, the honors of the Doctorate are conferred upon him; but he is not yet permitted to enter upon the practice of his profession. To enjoy this privilege, a more rigid ordeal must be passed; he is now called upon to demonstrate his *practical* acquaintance with the several subjects in which he has been instructed; to take charge of patients under the immediate inspection of his teachers, and finally to be questioned by eight of the most distinguished professors of the country in every department of medical science. By a recent decree of the University of France, it is now ordered that examinations shall be held at the termination of each year upon the branches which have been taught during the year; at the conclusion of the whole term of study, a final examination is instituted, embracing the subjects taught throughout the whole course. A knowledge of clinical medicine and surgery is also required, and a thesis must be publicly defended.

Although thus rapidly and most imperfectly enumerated, we have thought it not inappropriate to the subject to mention the requisitions which are demanded in foreign schools. They either exact of their pupils an unnecessary standard of requirements, or we, on the contrary, are satisfied with too low a grade of qualification. Which alternative is this convention prepared to adopt? Are we willing to assert, and maintain the opinion that he who has passed eight months within the walls of a medical college is as well fitted for the practical duties of his profession, as he who has devoted years to more extended studies? Assuredly we cannot advocate a proposition so absurd.

Nor, perhaps, is it at all requisite to occupy the time of the convention with a long detail of arguments in order to prove the *necessity* of some measures of reform.

In regard to the two subjects of preliminary education and the mode of examination, this committee has nothing to do. But being charged with the duty of reporting upon a uniform and elevated standard of requirements for the degree of M. D., whatever alteration in the present system it may recommend, will more immediately bear upon the interests of the schools. Yet let it not be supposed, as some we fear have imagined, that the object of this Committee, or of this convention, is to attack with a ruthless hand the institutions of the country, rendered venerable by time, or that still larger number whose charters have been obtained at a more recent date. We believe that no agrarian feeling has stimulated to action the advocates of reform in this or any other land: we dare express the conviction that the best interests of society and of the profession itself (which spring from no selfish considerations,) could alone have commenced a work, which, if calmly and perseveringly conducted, must redound to the good of all.

We are aware that many obstacles are opposed to the successful prosecution of the several matters upon which we have assembled to deliberate. With no National Legislature to regulate our Medical Institutions, and with a reckless indifference to their concerns too often practised by our State Governments, each has endeavoured to build up for itself, if not an enduring reputation, at least a long catalogue of names. And the colleges themselves, resting secure in their chartered rights, may contend against any interference with their several systems of education; although we would fain indulge the hope that some among them stand prepared to listen to that voice which rarely speaks in vain—the voice of public opinion. This it is which now agitates the professional mind from one end of this Union to the other; and this it is which, if not now heeded, will not cease to reiterate its cry. Let but a very few even of our conspicuous institutions demand of their pupils a more extended course of study, and so far from their interests perishing in the attempt, we cannot doubt that an accession to their numbers will ere long reward their efforts. The possession of the diploma no longer tests the qualification of the man; and it cannot be doubted that the large number of Medical Colleges throughout the country, and the facility with which the degree is obtained, have exerted a most pernicious influence. Numbers of young men have entered the profession who were not prepared for its onerous and responsible duties, who, after a longer or shorter period of disappointment, have served to swell the list of empirics, or to seek employment by means unbecoming the character of the physician or the gentleman. Nor can it well be otherwise, when each returning spring lets loose upon the community some twelve or thirteen hundred graduates, whose professional existence must depend upon the encouragement they receive.

To correct these evils it is indispensable that the standard both of preliminary and of medical education should be elevated; and as we have said before, let no institution fear to commence the laudable undertaking. The encouraging plaudits of the profession will cheer it on, and its general voice will direct our medical students to that source of information from which is to be derived the largest supply. We are, moreover, unwilling to inflict such discredit upon the ingenuous youth of the land as to imagine, that they would not voluntarily become the pupils of that school whose diploma is to be gained by a more prolonged and complete course of study. They will feel that their success in after life will mainly depend upon the labours of their earlier years; and when success shall crown their efforts, they will enjoy the proud satisfaction of knowing that it is based, not upon the disgraceful trickery of the impostor, but upon the solid foundation of positive acquirement.

It may not, perhaps, be wandering too far from our legitimate course to remark, that no ordinary responsibility rests upon Professors themselves. They stand as the guardians, more than any others, of the public weal; for every student who receives the diploma, is furnished by them with *prima facie* evidence, that he is prepared for that important occupation in which he seeks to engage. And if we admit the supposition that in any case he is not thus prepared, it will require the prediction of no seer to foretell the lamentable result. We believe the opinion defensible, that in no profession is it more difficult to arrive at just conclusions, as to real merit, than in that of medicine. "And hence (in the language of another) there devolves upon those who are the most competent to judge, and who have the best opportunity for judging, a vast responsibility to see well to it that none but the truly meritorious should be admitted to the profession under their sanction. From the fact that society cannot in this respect adequately protect itself, the Teachers in our Medical Schools should exercise a guardianship the more watchful, a jealousy the more keen, and a firmness the more unyielding." With a determination thus expressed, to furnish the community with individuals who have gained the honours of the Doctorate solely on account of their merit, it cannot be saying too much when we assert that a reciprocal duty rests upon it; a duty which should ever lead to the protection and encouragement of those who have devoted their time and talents to its dearest interests.

The most beneficial consequences may also be expected from the efforts of private Preceptors. The position can scarcely be controverted, that a proper inquiry into the fitness of young men to become Students of Medicine is but seldom instituted; and numbers have been admitted into the offices of their Preceptors who were deficient even in the most common branches of an English education. Too long has it been imagined, and too often has it been asserted, that the veriest dolts possessed intellect sufficient for the study of medicine. To such the idea could never have occurred, that within its broad and expanded limits may be profitably included a knowledge of almost every science ; nay, more, may we not truly say that a high and exalted position can never be attained, unless the mind be well stored with an amount of collateral information which will be found to bear upon our professional studies at every step ?

There is another point in which the preceptor sometimes fails to do his duty. We are too often satisfied with merely placing the most approved authors, if you please, in the hands of our pupils, without instituting a regular series of examinations and explanations, in default of which the impressions produced by reading are frequently evanescent, if not erroneous. The inexperienced mind of the student requires so to be regulated in its pursuits, that each successive link in the chain of information may be properly adjusted ; by which arrangement not only is much economy of time and labour secured, but the field of knowledge is thus gradually expanded before him, until finally he is brought to comprehend its whole area in all its uniformity and beauty. Let us then exercise a jealous watchfulness over our pupils in these particulars ; let us remand to academical teachings the individual who has neglected the rudiments of a common education, and let us feel that we have not rightfully earned our fees of tuition, unless we shall have honestly laboured to advance the student's interests by all the means in our power.

In entering upon the principal purport of this report, it is deemed advisable to remark, that in order to obtain a knowledge of the requirements demanded by the various schools of the country, a letter was addressed to all whose existence was known to your committee, requesting them to furnish the desired information. These consisted of thirty-three in number ; and answers were received from nineteen, being a fraction more than one-half. In the majority of instances pamphlets were forwarded to the chairman, containing the rules and regulations of the college, together with the branches taught ; while from others, letters were received simply stating that two full courses of lectures were demanded, that the candidate must be twenty-one years of age, of good moral character, &c. From this correspon dence but little available information was derived. A circular was then dispatched to the different colleges propounding the following questions, answers to which were respectfully solicited. Nineteen of these circulars were filled up and returned.

State 1*st*. The number of students during the session of 1845–6.
2d. The number of graduates in 1845–6.
3*d*. The number of charity pupils in 1845–6.
4*th*. The number of professors attached to your school.
5*th*. The date at which lectures begin and end.
6*th*. The requirements for a degree.
7*th*. Is the inquiry made previous to examination, whether or not these requirements are fulfilled ?
8*th*. Is any evidence of having attended a course of clinical instruction necessary to graduation ?

9*th*. Is it required of candidates for graduation that they shall have devoted any time to dissection?

The responses to these several questions have been arranged in tabular form, from which the result of the whole correspondence may be ascertained at a glance, and which your committee beg leave to lay before this Convention, as a part of this report. We cannot avoid the expression of our regret, that so many of the colleges have failed to respond to our circular, not, we hope, from a listless indifference to the subject, or a determination not to abide by the recommendations of this body. Information was also requested from the proper authorities in regard to the requirements of surgeons in the army and navy of the United States; but no reply has been received from them. (See Note A.)

In summing up the information obtained from the nineteen colleges named in the table, in the order in which the questions were proposed, it will be found that the number of students belonging to these institutions during the session of 1845–6, amounted to 2544, including 31 non-paying pupils; and that the number of graduates was 730;—the number of professors varies from *three* to *eight*. It may be proper to remark that the lowest number here named is attached to the University of Virginia, where the usual branches of medical education are taught, and where the term of study extends throughout the period of *nine* months. If we exclude this institution from our table, the minimum is *five* and the maximum is *eight*. The time employed in lecturing also varies; *thirteen* weeks being the shortest, and *eighteen* the longest period. *Sixteen* weeks, however, are devoted to the lectures, in a large majority of the schools. The requirements for the degree of M. D. appear to be very general;—the candidate must be twenty-one years of age;—his moral character good;—his examination satisfactory;—his thesis passable, and he must have attended two full courses of lectures, to be included within the period of three years' study. In some of the schools a practice of four years and an attendance on one course of lectures, is sufficient to entitle the individual to an examination. Branches are taught in some institutions which are omitted in others, and the manner in which these are distributed is by no means uniform. It appears without exception that the inquiry is made previous to examination, whether or not all the requirements have been fulfilled, and in some cases unquestionable proof of the fact must be adduced. Evidence of having attended a course of clinical instruction is required in *twelve* of the colleges, while in *seven* it is not; and as to dissection, *five* render it obligatory, and the remaining *fourteen* are content to urge its recommendation.

Such, then, is the information which the committee, after diligent inquiry, is enabled to lay before this body. It would have been a matter of some importance to have received answers from all the schools, for in that case an account entirely accurate of the whole number of students in the United States would have been obtained. (See Note B.)

It will be seen that the nineteen colleges which noticed our circular, report the aggregate number to be 2544; and if it be proper to assign the same ratio to the fourteen which did not, the whole number of students during the session of 1845–6, will be found to be 4418 and a fraction. We believe this to be a very fair statement, for several statistical tables published from time to time in the journals, have varied but little from this amount; and if we extend the same rule to the classes of graduates, we reach the conclusion that a fraction less than 1300 were added to the already existing number of physicians, in the spring of 1846.

The very large number of physicians in the United States, a number far larger in proportion to its population than in any other country perhaps of which we have a correct knowledge, has frequently been the subject of remark. To relieve the diseases of something more than twenty millions of people, we have an army of Doctors amounting by a recent computation to forty thousand, which allows one to about every five hundred inhabitants. And if we add to the 40,000 the long list of irregular practitioners who swarm like locusts in every part of the country, the proportion of patients will be still further reduced. No wonder, then, that the profession of medicine has measurably ceased to occupy the elevated position which once it did; no wonder that the merest pittance in the way of remuneration is scantily doled out even to the most industrious in our ranks,—and no wonder that the intention, at one time correct and honest, will occasionally succumb to the cravings of a hard necessity. The evil must be corrected. With a government like ours, to diminish the number of medical schools is not to be expected; and the corrective can alone be found in the adoption of such a standard of requirement, in the general estimate of which the recommendations of this committee will form but an item, as will place the diploma beyond the reach of those who seek to wear its honours without deserving them.

The shortness of the time allotted to the delivery of Lectures, we believe to be an evil of no small magnitude. It is next to an impossibility, that the strongest intellect can receive and well digest some half a dozen discourses or more a day, embracing subjects which have oftentimes little or no immediate connection with each other. The mind becomes wearied with the multiplicity of its occupations, and the thoughts of to-day are forgotten in the constantly recurring and harassing duties of the morrow. A proper allotment of time cannot be given to that deep reflection which the importance of the subject demands, and without which no solid advancement can be made. There are individuals, too, who do not always rest satisfied with professorial prelections, who approve in its fullest extent the adage, "nullius addictus jurare in verba magistri," and who seek in the recorded experience of others the widest extent of information. Nor can Teachers themselves, with every disposition to impart the fullest instruction, do justice to their several branches within the limited period of four months. For this reason, the examination which may, indeed, test the actual acquirement of the student, can never embrace an extensive field of knowledge. Under the present regulation this cannot be expected; for justice to the pupil must prevent an inquiry into subjects in which he has never been instructed.

In regard to the branches to be taught, it is not improbable that the recommendation of the Committee may not meet the views of some of the members of this Convention. It may be thought that others should have been added, and that consequently the number of Professors should have been considerably augmented. This subject was freely canvassed by the Committee, and the opinion was general that in the commencement of our effort to reform the existing condition of things, too high a standard of requirement ought not to be immediately insisted on. The conclusions to which this Convention may come upon any subject, cannot be forced upon the schools contrary to their wishes. In the advice of this body there exists, however, a moral power which it would be unwise to disregard, a power which, while it seeks to elevate medical requirement, should not impose a single requisition which is not entirely practicable. Besides, it is to be recollected that the decrees of this Convention need not be final and unalterable. The National Medical Association, contemplated under the first two resolutions adopted at the meeting in May last, which it is to be hoped will convene from time to time in different parts of the country, can legitimately exercise the same influence which we now seek to do; and when in after time it may be deemed advisable to add new subjects of instruction, it will not hesitate to express its opinion. Under this view of the matter, your Committee advise that lectures be given on the following branches, in all the Colleges: viz., on the Theory and Practice of Medicine; the Principles and Practice of Surgery; General and Special Anatomy; Physiology and Pathology; Materia Medica; Therapeutics and Pharmacy; Midwifery, and Diseases of Women and Children; Chemistry and Medical Jurisprudence.

These subjects, it is true, are now taught in the majority of our schools. With a lengthened period for teaching, a double advantage will be gained; a wider extent of information may be imparted to the student, while his time will be occupied with fewer lectures during the day. As to the appropriation of the several branches, it is deemed inexpedient to make any suggestion, as the circumstances of each Institution may be such as will necessarily lead to a different arrangement in this particular.—It is believed, however, that a slight augmentation in the number of Professors will become necessary.

In regard to clinical instruction, your Committee would willingly have made it a requisition to the attainment of the diploma; but an insuperable difficulty seemed to exist. It is believed that few of the Colleges have Hospitals attached to them, and in very many instances where such Institutions exist in the Cities, the Professors do not receive the appointment of attending Physicians and Surgeons. Consequently, the opportunity is lost to them of imparting such instruction as would be valuable to the student.

We cannot, however, close this portion of our report, without urging the very great importance of clinical instruction. Many a young man has entered upon the duties of his profession, sufficiently instructed, it may be, in its principles, who has most painfully felt the responsibility of his position. In default of a certain degree of practical knowledge, indecision must often fetter his efforts to the detriment of his patient, and that self-confidence, which can alone restore serenity to his mind, must be purchased by a long period of perplexing doubt. During the pupilage of the student, he should lose no opportunity of witnessing cases of disease wherever they are to be found. Instructors themselves, should esteem it their highest duty to introduce their pupils to the bedside of their patients in all cases where propriety will admit of it. The Hospitals, Penitentiaries, and Poor-houses, found in so many of our towns and cities, will furnish from time to time, cases of every description, while the pauper practice of the country will prove no indifferent means of imparting clinical information. Instruction gained from these sources in free and familiar conversation with the Preceptor, will perhaps be of more avail than any other; for the large number of students who occasionally throng the Wards of the Hospital, render it impossible for each one to obtain a correct knowledge of the cases presented to him. We do not intend by this remark to convey the idea that information given in this way would be valueless. On the contrary, when properly conducted, it may be made productive of the greatest good. Let the Professor daily analyze the symptoms which arise in every case; have them carefully noted down in his ward-book, together with his prescriptions, and at its termination let him review the whole ground, aided by a post-mortem examination if it result unfavourably, and he will impart instruction which is truly valuable.

By reference to the table accompanying this report, it will be seen that only five of the colleges insist upon dissection as a requisite to graduation. Your committee accord in the opinion, that all should render it obligatory upon the student to devote some portion of his time to this needful pursuit. To enter into an argument to prove its absolute necessity not only to the surgeon but also to the physician, would be a work of supererogation. We therefore offer without further remark, the following resolutions for the adoption of this Convention:

*Resolved*, 1st. That it be recommended to all the colleges to extend the period employed in lecturing, from four, to six months.

2d. That no student shall become a candidate for the degree of M. D., unless he shall have devoted three entire years to the study of medicine, including the time allotted to attendance upon the lectures.

3d. That the candidate shall have attended two full courses of lectures, that he shall be twenty-one years of age, and in all cases shall produce the certificate of his preceptor, to prove when he commenced his studies.

4th. That the certificate of no preceptor shall be received who is avowedly and notoriously an irregular practitioner, whether he shall possess the degree of M. D. or not.

5th. That the several branches of medical education already named in the body of this report, be taught in all the colleges ; and that the number of Professors be increased to seven.

6th. That it be required of candidates that they shall have *steadily* devoted three months to dissections.

7th. That it is incumbent upon Preceptors to avail themselves of every opportunity to impart clinical instruction to their pupils ; and upon Medical Colleges to require candidates for graduation to show that they have attended upon Hospital practice for one Session, whenever it can be accomplished, for the advancement of the same end.

8th. That it be suggested to the Faculties of the various Medical Institutions of the country to adopt some efficient means for ascertaining that their students are actually in attendance upon their lectures.

9th. That it is incumbent upon all schools and colleges granting Diplomas, fully to carry out the above requisitions.

10th. That it be considered the duty of Preceptors, to advise their students to attend only such institutions as shall rigidly adhere to the recommendations herein contained.

All which is most respectfully submitted.

*May*, 1847. Ro. W. Haxall, *Chairman.*

Note A.—After finishing this report, a communication was received from H. L. Heiskill, acting surgeon-general, in answer to the letter addressed to the surgeon-general late in May, 1846.

The absence of this officer from the seat of government prevented an earlier reply. Such portions of said communication as have reference to the subject matter of this report, your committee have thought proper to embody in this note. Many of the duties required of army surgeons are of course not performed by practitioners in civil life.

"Under the regulations the medical officer is required to investigate the physical agents that may affect the health of the troops, to make reports upon the medical topography of his station, to aid in the selection of military positions, and, as a question of military hygiene, to express his views in respect to the diet, clothing, watering and exercises of the troops. He is from the necessity of the case a *general practitioner* in the most extended sense of the term, and from location he is often thrown entirely upon his own resources, and deprived of the advantage of calling to his aid the friendly counsel of professional brethren."

"Nor have the army medical officers been regardless of the high claims of medicine in itself as one of the *liberal* professions. Them selves medical men, they feel a deep interest in the character of the profession, and rejoice in any measure that would tend to promote its honour and efficiency."

Next follows a table showing the number of applicants examined from the year 1841 to 1845, both inclusive, being a period of five years. It here appears that 51 were examined, while only 17 were approved.

"The most striking cause of failure on the part of the candidates are, *insufficient preparatory education; a hurried course of professional pupilage; want of proficiency in practical anatomy*, in *pathology*, and in *clinical medicine*."

"The candidates are examined on the branches usually taught in our medical schools, also on their literary and scientific attainments, embracing a grammatical knowledge of the English language, Latin, and natural philosophy (or physics,) which are deemed important branches of education to those entering on the study of a *liberal* profession. Each candidate is required, as introductory to his examination, to prepare in writing a brief extemporaneous description of the causes, symptoms, pathology, treatment, &c., of such diseases as may be assigned by the Board, together with one or more prescriptions proper in the case, written out in form for the apothecary."

A merit roll accompanies the communication, which shows the branches upon which the applicants are examined. These are as follows: "Literary and Scientific qualifications, Anatomy and Physiology, Principles and Practice of Surgery, Principles and Practice of Medicine, Obstetrics, Materia Medica, Chemistry and Medical Jurisprudence."

"In the several branches herein enumerated, the candidate is required to be well grounded. The examinations are conducted with the view of ascertaining the measure of his natural endowments, his general professional intelligence, as well as his exact knowledge, his practical ability and strength of judgment."

Note B.—The Chairman received the reply of the Indiana Medical College, located at Laporte, on the Thursday before leaving home;—too late to be inserted in the table which had then been completed. In 1845–6, it had 81 students, 18 graduates and 6 Professors.

### TABLE.

| Names of Medical Colleges. | Number of students in the Session of 1845–6. | Number of graduates at close of session of 1845–6. | Number of charity pupils in 1845–6. | Number of professors attached to the college. | Date at which the lectures begin and end. | The requirements for the degree of M. D. | Is inquiry made before examination if they are fulfilled? | Is clinical instruction necessary to graduation? | Is dissection required as indispensable to graduation? |
|---|---|---|---|---|---|---|---|---|---|
| Medical College of Georgia. | 112 | 30 | 0 | 7 | 2d Monday in November. Last day of February. | 2 full courses of lectures. Usual private reading. Thesis. Good character. | Yes | No | Yes |
| Medical School of Me. Brunswick, Vermont. | 70 | 19 | 0 | 5 | Middle of Feb., and continue 13 weeks. | 3 years study. 2 courses of lectures. Good character. | Yes, and enforced | No | No |
| Medical Department of the University of St. Louis. | 53 | 11 | 0 | 7 | 1st Monday in Nov., end 1st day of March. | 3 years study. 2 courses of lectures. 21 years old. Thesis. | Yes | Yes | No |
| Dartmouth College, Hanover, N. H. | 85 | 31 | 0 | 6 | Aug. 13., Nov. 21, 16 weeks. | 2 courses of lectures. 3 full years study. Thesis. | Written evidence to be placedon file | No | No |
| Willoughby Medical College. | 164 | 32 | 0 | 8 | 1st Wednesday in Nov., continue 16 weeks | 3 years study. 2 courses of lectures. 21 years old. Thesis. | Yes | No | No |
| Medical College of Ohio, Cincinnati. | 195 | 46 | 3 | 6 | 1st Monday in Nov. Last day of February. | 21 years. 3 years study. 2 courses. | Certificate required to prove it | No | No |
| University of Pennsylvania. | 414 | 171 | 6 | 7 | 1st Monday in Nov. About middle of March. | 21 years. 3 years study. 2 courses of lectures. Thesis, &c. | Tickets are always examined | Yes | No |
| Franklin Medical College, Philadelphia. | 35 | | - | 7 | 2d Monday in Oct. Last day of February. | 3 years study. 2 courses. 21 years, &c. | Yes | Always | No |
| Medical Department of University of Missouri. | 85 | 9 | 0 | 6 | 1st Nov. 1st March. | 2 y'rs. study and 2, or 3 courses, or 1 course with 3 years practice. | Yes | Yes | Yes |

| | | | | | | | | |
|---|---|---|---|---|---|---|---|---|
| Medical Department of University of Louisville. | 345 | 73 | 0 | 7 | 1st Monday in Nov. Last day of February. | 2 courses, 21 years; or 1 course and 4 years practice. | Yes | Yes | Yes |
| Geneva Medical College, New York. | 178 | 45 | four state pupils | 6 | 1st Monday in Oct. Last Tuesday in Jan. | 21 years. 2 courses. Thesis. | Written evidence or sworn to | No | No |
| Massachusetts Medical College. | 159 | 47 | 0 | 6 | 1st Wednesday in Nov. 1st Wednesday in March. | 21 years. 2 courses. 3 years. | Yes | Yes | No |
| Albany Medical College, New York. | 113 | 46 | four by law | 8 | 1st Tuesday in Oct. 16 weeks. | 21 years. 3 years study. 2 courses. Thesis. | Certificates are required | No | No |
| Western Reserve College, Cleveland, Ohio. | 160 | 53 | 0 | 7 | 1st Monday in Nov. 16 weeks. | 3 y'rs. study. Thesis. 2 courses, or 4 years practice and 1 course. | Yes | No | No |
| University of Virginia. | 55 | 13 | | 3 | Continue 9 months. | • | | | |
| Medical Institution of Yale College, N. Haven. | 53 | 19 | 8 | 6 | Information is imperfect. | Bachelors of arts, 2 y'rs. study, all others 3 y'rs. 2 courses. 21 y'rs. &c | Certificates are required | No | No |
| College of Physicians and Surgeons, N. Y. City. | 218 | 48 | 4 | 6 | 1st of November. Last day of February. | 21 years. 3 years study, 2 courses, First examination before faculty, second before trustees, third before public on Thesis. | Certificates are required | No | No |
| Med. Department of Hampden Sydney College, Richmond, Va. | 74 | 18 | 0 | 6 | 1st of November. 1st of March. | 2 courses of lectures. 21 years. Thesis, &c. | Yes | No | Yes, one course |
| Washington University of Baltimore. | 45 | 19 | 2 | 7 | 1st of November. 1st of March. | 2 courses of lectures. Thesis, &c. | Yes | No | Yes |
| Total . . . Non-paying students, or charity students . . | 2513<br>31<br>2544 | 730 Graduates | 31 non-paying students (charity) | | | | | | |

